# Calorie Restriction Provides Kidney Ischemic Tolerance in Senescence-Accelerated OXYS Rats

**DOI:** 10.3390/ijms232315224

**Published:** 2022-12-03

**Authors:** Nadezda V. Andrianova, Ljubava D. Zorova, Irina B. Pevzner, Nataliya G. Kolosova, Egor Y. Plotnikov, Dmitry B. Zorov

**Affiliations:** 1 Belozersky Institute of Physico-Chemical Biology, Lomonosov Moscow State University, 119992 Moscow, Russia; 2 Kulakov National Medical Research Center of Obstetrics, Gynecology, and Perinatology, 117997 Moscow, Russia; 3 Institute of Cytology and Genetics, Siberian Branch of Russian Academy of Sciences (SB RAS), 630090 Novosibirsk, Russia

**Keywords:** aging, ischemia/reperfusion, kidney injury, therapy, calorie restriction, mitochondria

## Abstract

Kidney diseases belong to a group of pathologies, which are most common among elderly people. With age, even outwardly healthy organisms start to exhibit some age-related changes in the renal tissue, which reduce the filtration function of kidneys and increase the susceptibility to injury. The therapy of acute kidney injury (AKI) is aggravated by the absence of targeted pharmacotherapies thus yielding high mortality of patients with AKI. In this study, we analyzed the protective effects of calorie restriction (CR) against ischemic AKI in senescence-accelerated OXYS rats. We observed that CR afforded OXYS rats with significant nephroprotection. To uncover molecular mechanisms of CR beneficial effects, we assessed the levels of anti- and proapoptotic proteins of the Bcl-2 family, COX IV, GAPDH, and mitochondrial deacetylase SIRT-3, as well as alterations in total protein acetylation and carbonylation, mitochondrial dynamics (OPA1, Fis1, Drp1) and kidney regeneration pathways (PCNA, GDF11). The activation of autophagy and mitophagy was analyzed by LC3 II/LC3 I ratio, beclin-1, PINK-1, and total mitochondrial protein ubiquitination. Among all considered protective pathways, the improvement of mitochondrial functioning may be suggested as one of the possible mechanisms for beneficial effects of CR.

## 1. Introduction

The population of elderly people in the world is constantly increasing, and currently, the number of people being 65 and older reached more than 650 million (9% of the world population) [1]. Older age is one of the risk factors of acute kidney injury (AKI) characterized by a reduced glomerular filtration rate and rapid accumulation of metabolic waste products [2]. Elderly patients have a greater risk of developing AKI, higher mortality, and a longer recovery period after renal injury [3]. With age, the likelihood of developing AKI complications, notably the transition of AKI to chronic kidney disease (CKD) increases manyfold [4]. The mortality rate of the elderly patients with acquired AKI is reported to be around 57% which is alarming and requires additional public attention to this pathology [5].

Despite the variety of causes of AKI, ischemic injury is suggested to be one of the main damaging factors [6]. Most often, ischemic AKI occurs in patients with cardiovascular disease or after surgery, as well as in kidney transplant recipients [7]. High mortality from AKI, including of ischemic nature, may be associated with the absence of pharmacologic treatment of AKI since currently there are no targeted pharmacotherapies approved for this pathological condition [8]. Therefore, in most cases of AKI, treatment is limited exclusively to renal replacement therapy and hemodynamic support [9]. In this regard, there is a need to develop more specific and practical approaches for AKI therapy.

One of the approaches that looks promising in therapeutic use against AKI is calorie restriction (CR) [10]. CR is defined as a reduction of food or the consumption of certain nutritional elements with no apparent signs of malnutrition [11]. CR both significantly increases life expectancy and delays the development of age-related diseases, including renal diseases [12]. Positive effects of CR are mediated through several signaling pathways, typically resulting in a significant rearrangement of cell metabolism, activation of autophagy, and improving the functioning of mitochondria [13]. Activation of these signaling pathways may contribute to an increase in tissue tolerance to damage. In this regard, using CR as an approach for AKI therapy deserves special consideration [14].

Since the renal tissue of elderly patients is characterized by some morphological and functional peculiarities [15], it is advisable to develop and test AKI therapy on animals with corresponding changes in the renal tissue. However, experiments on old animals are accompanied by extensive material and time costs. The special animal strains have been bred with senile changes appearing already at a young age. One of these strains is senescence-accelerated OXYS rats.

The OXYS strain was derived from the Wistar rats via selection for susceptibility to cataractogenic effects of a galactose-rich diet as described earlier [16]. Then, it has been discovered that OXYS rats spontaneously develop accelerated-senescence syndrome, as early manifestation of a phenotype similar to human geriatric disorders including cataract, involution of the thymus, hypertrophic cardiomyopathy, sarcopenia, osteoporosis, AMD-like retinopathy, and signs of Alzheimer’s disease [17,18,19,20,21,22]. Phenotypic manifestations of all these pathologies occur at the age of 3 months and then gradually accelerate. In addition, some parameters, including the levels of oxidative stress, NO production, mitochondrial functioning, autophagy machinery, and gene expression corresponded to those of old rats [23,24,25,26,27,28,29]. The kidney functioning of OXYS rats and the response to ischemic preconditioning were also more corresponding to old animals than young ones [30].

Based on this, it is assumed that OXYS rats can serve as a reliable animal model for studying the molecular mechanisms of aging and age-related diseases. In this study, we analyzed the potential nephroprotective effects of CR in OXYS rats. Such data on aged kidney tissue is very limited making these results of great scientific interest. Moreover, we did not limit our study to the physiological effects of CR, but rather tried to unravel possible molecular mechanisms of nephroprotection provided by CR in senescence-accelerated OXYS rats. For this purpose, we analyzed some parameters related to renal injury, recovery response to injury, cellular senescence, the functioning of mitochondria, mitochondrial dynamics, the activation of autophagy and mitophagy, and the outcome of oxidative stress.

## 2. Results

### 2.1. CR Causes Significant Weight Loss in OXYS Rats

**Figure 1:**
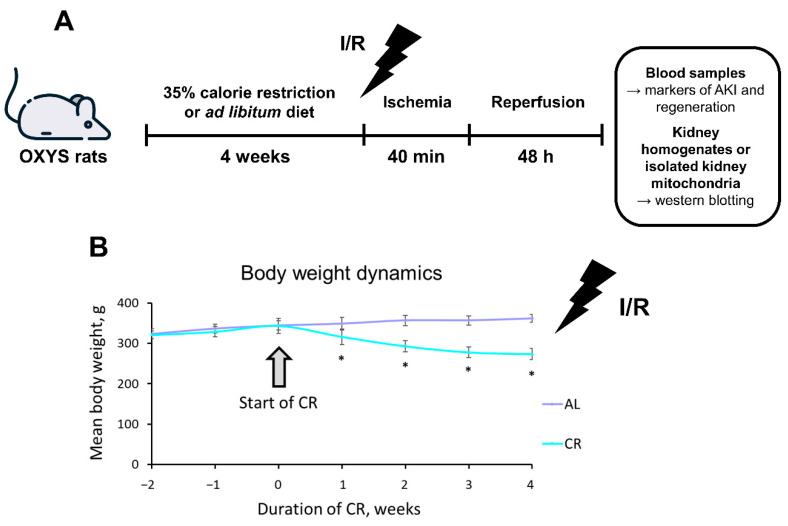
Experimental design and body weight dynamics during the study. (**A**) Experimental design included dietary protocol 4 weeks prior to ischemia following by reperfusion with further collecting samples; (**B**) Weight changes in AL (*n* = 11) and CR (*n* = 10) groups of OXYS rats during the experiment. * *p* < 0.05 (Mann–Whitney U-test).

During the entire study, weekly, the weight of OXYS rats in all groups (*n* = 11 for AL group and *n* = 10 for 35% CR group) was measured (Figure 1). For the first 2 weeks, the normal ad libitum (AL) food intake was assessed, which allowed us to accurately calculate the required amount of food for the CR group. The amount of food consumed AL or during CR is listed in the Dietary protocol of the Methods section. Reduction of consumed food for 35% during CR resulted in significant body weight loss in OXYS rats (Figure 1B). Whereas each rat on the AL diet gained approximately 17 g (5% of the initial body weight) during 4 weeks, each rat on CR lost nearly 70 g (20% of the initial body weight). At the end of the experiment, the total difference of mean body mass between AL rats and rats on CR was 87 g.

### 2.2. CR Reduces AKI Severity in OXYS Rats

**Figure 2:**
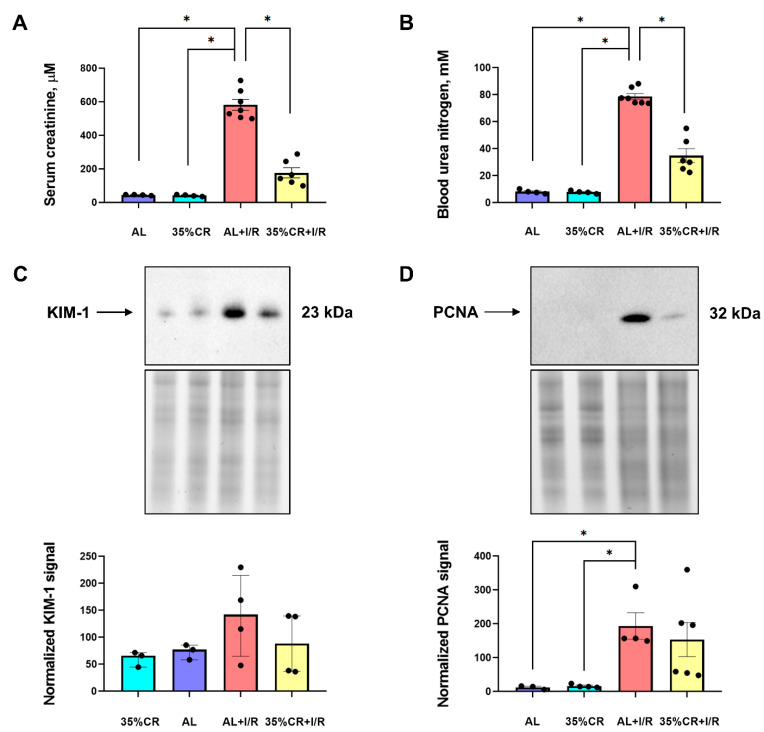
Assessment of renal function and regeneration rate after renal I/R in OXYS rats. (**A**) The severity of AKI measured by serum creatinine concentration 48 h after I/R in OXYS rats on 35% CR or AL (*n* = 4 for AL group, *n* = 4 for 35% CR group, *n* = 7 for AL+I/R group, *n* = 6 for 35% CR+I/R group); (**B**) The severity of AKI measured by blood urea nitrogen concentration 48 h after I/R in OXYS rats on 35% CR or AL (*n* = 4 for AL group, *n* = 4 for 35% CR group, *n* = 7 for AL+I/R group, *n* = 6 for 35% CR+I/R group); (**C**) KIM-1 levels in kidney homogenates of OXYS rats on 35% CR or AL diet, before and 48 h after renal I/R (*n* = 3 for 35% CR group, *n* = 3 for AL group, *n* = 4 for AL+I/R group, *n* = 4 for 35% CR+I/R group); (**D**) PCNA levels in kidney homogenates of OXYS rats on AL diet, before and 48 h after renal I/R (*n* = 3 for AL group, *n* = 4 for 35% CR group, *n* = 4 for AL+I/R group, *n* = 6 for 35% CR+I/R group). Below the blot image of the target protein, the corresponding total protein loading estimated by the stain-free imaging technique is presented (**C**,**D**). * *p* < 0.05 (one-way ANOVA).

To determine the severity of kidney injury, we assessed the accumulation of serum creatinine (SCr) and blood urea nitrogen (BUN) 48 h after renal ischemia/reperfusion (I/R) (*n* = 7 for AL+I/R and *n* = 6 for CR+I/R groups) (Figure 2A,B). An increase in the content of these end-products of nitrogen metabolism usually indicates a deterioration of the renal filtration and excretory functions. In this regard, SCr and BUN are widely used markers of various kidney diseases [31]. In intact animals, 35% CR itself did not change SCr (44.0 ± 2.0 μM in AL group and 41.3 ± 2.7 μM in CR group) or BUN concentrations (8.2 ± 1.1 mM in AL group and 7.9 ± 0.5 mM in CR group) in serum (Figure 2A,B). In OXYS rats kept on AL diet, I/R led to a 9-fold increase in BUN and a 13-fold increase in SCr concentrations. However, OXYS rats subjected to 35% CR for 4 weeks before I/R showed less pronounced increase in nitrogenous waste compounds. The SCr concentration after I/R elevated to 581.6 ± 86.1 μM in OXYS rats kept on AL diet, whereas in OXYS rats on CR it was 224.6 ± 144.4 μM. The BUN level was 78.7 ± 5.9 mM in the AL+I/R group with 40.2 ± 18.2 mM in the 35% CR+I/R group. Thus, CR contributed approximately to a 2-fold decrease in these markers of renal function in OXYS rats, indicating the reduced severity of I/R-induced AKI in animals kept on CR (Figure 2A,B).

In addition to SCr and BUN, which are conventional markers mostly reflecting renal failure, we analyzed the levels of kidney injury molecule-1 (KIM-1) (Figure 2C). KIM-1 is suggested to be a biomarker of AKI at an early stage and to be more sensitive than SCr and BUN [32]. We found that KIM-1 levels tended to increase after I/R, while CR prevented kidney injury returning KIM-1 level to the values of control rats (Figure 2C).

We have also evaluated the dynamics of proliferating cell nuclear antigen (PCNA), which is one of the most used markers of proliferation and regeneration in clinical and experimental practice [33]. In kidney homogenates, a significant increase in PCNA level was found in rats 48 h after I/R compared to intact animals kept on the same diet (Figure 2D). The PCNA level rose in kidneys after injury 19 times in rats on the AL diet and approximately 16 times in the CR group. In the serum of OXYS rats, we measured the levels of growth differentiation factor 11 (GDF11), which participates in development and regeneration of the kidney [34] and demonstrated anti-aging effects for various organs in old animals [35,36,37]. The levels of GDF11 precursor at 50 kDa demonstrate slight increase in serum after CR, while the mature peptides of GDF11 at 13 kDa did not change in serum in response to CR, and dropped after I/R in both CR and AL groups (Supplementary Figure S1).

### 2.3. CR Affects Apoptotic Pathways and the Level of Oxidative Stress in OXYS Rats

It is believed that mitochondria are involved in the majority of pathological effects of I/R injury [38]. In addition, mitochondria are one of the most critical targets of CR [11]. These facts lead to the conclusion that the functioning of mitochondria is essential for ischemic tolerance development and may be a participant in the protective signaling pathway of CR. In this study, we analyzed the functions of mitochondria by evaluating the content of Bcl-2 family proteins and the level of oxidative stress indexed by the protein carbonylation.

The Bcl-2 family, which includes more than 30 proteins, contains both proapoptotic and antiapoptotic proteins [39]. Their ratio and interaction with other proteins determine the possibility of permeabilization of mitochondrial membranes and the development of an apoptotic response. Among proteins of this family, we measured levels of the anti-apoptotic protein Bcl-X_L_ and the pro-apoptotic Bcl-X_S_, which is formed from the Bcl-X_L_ as a result of alternative splicing.

**Figure 3:**
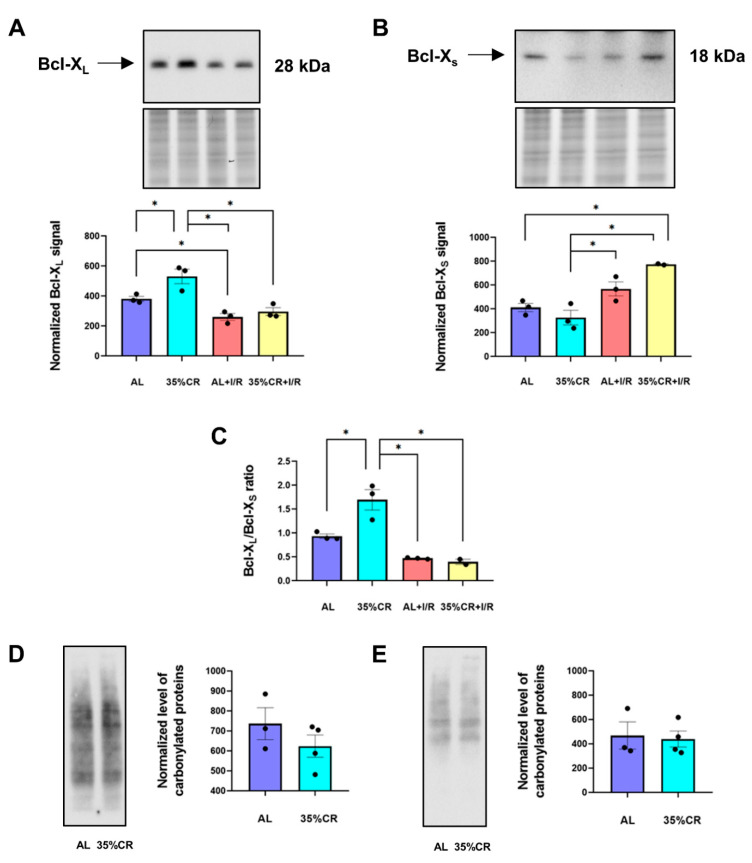
Changes in anti- and pro-apoptotic proteins and the level of protein carbonylation. (**A**) Bcl-X_L_ levels in isolated kidney mitochondria of OXYS rats kept on either 35% CR or AL diet, before and 48 h after renal I/R (*n* = 3 for AL group, *n* = 3 for 35% CR group, *n* = 3 for AL+I/R group, *n* = 3 for 35% CR+I/R group); (**B**) Bcl-X_S_ levels in isolated kidney mitochondria of OXYS rats on 35% CR or AL diet, before and 48 h after renal I/R (*n* = 3 for AL group, *n* = 3 for 35% CR group, *n* = 3 for AL+I/R group, *n* = 2 for 35% CR+I/R group); (**C**) Bcl-X_L_/Bcl-X_S_ ratio in isolated kidney mitochondria of OXYS rats on 35% CR or AL diet, before and 48 h after renal I/R (*n* = 3 for AL group, *n* = 3 for 35% CR group, *n* = 3 for AL+I/R group, *n* = 2 for 35% CR+I/R group); (**D**) The level of protein carbonylation in kidney homogenates of OXYS rats on 35% CR or AL diet (*n* = 3 for AL group, *n* = 4 for 35% CR group); (**E**) The level of protein carbonylation in kidney mitochondria of OXYS rats on a 35% CR or AL diet (*n* = 3 for AL group, *n* = 4 for 35% CR group). Below the blot image of the target protein, the corresponding total protein loading estimated by the Stain-Free imaging technique is presented (**A**,**B**). * *p* < 0.05 (one-way ANOVA).

In our study, we observed that I/R significantly decreased Bcl-X_L_ levels and tended to increase Bcl-X_S_ in isolated kidney mitochondria (Figure 3A,B), indicating deteriorating effects of I/R. On the contrary, 35% CR for 4 weeks elevated Bcl-X_L_ in OXYS rats (Figure 3A). Since apoptotic response and cell survival depends on anti-apoptotic/pro-apoptotic Bcl-X_L_/Bcl-X_S_ ratio rather than the individual levels of these proteins, we also calculated this ratio in kidneys of OXYS rats (Figure 3C). We observed that CR significantly increased Bcl-X_L_/Bcl-X_S_ ratio in isolated kidney mitochondria indicating pro-survival state of the kidney after CR. Of note, since Bcl-X_L_ and Bcl-X_S_ may localize not only in mitochondria, but also in the endoplasmic reticulum [40], we also detected the levels of these isoforms in kidney homogenates (Supplementary Figure S2). The levels of Bcl-X_L_ and Bcl-X_S_ measured in kidney tissue showed a similar shift as observed in isolated mitochondria.

The severity of oxidative stress is directly associated with the change in the functioning of mitochondria and results in excessive production of reactive oxygen species (ROS) over the buffering capacity of endogenous antioxidant defense. Assessment of oxidative stress is essential for studies of aging and age-related diseases. One of the most widely used methods for evaluating the degree of oxidative changes is the level of protein carbonylation [41]. Carbonyl modifications can be generated by a variety of free radicals as well as by-products of lipid oxidation [42]. In OXYS rats, 35% CR for 4 weeks did not affect total protein carbonylation both in kidney homogenates (Figure 3D) and in isolated kidney mitochondria (Figure 3E).

### 2.4. CR Leads to Metabolic Shifts after I/R in OXYS Rats

The deficiency of oxygen and nutrients during I/R leads to a decreased energetic reliance on mitochondria, and forced activation of glycolysis [43]. In this regard, we measured the levels of cytochrome c oxidase (COX IV) in isolated kidney mitochondria and glyceraldehyde 3-phosphate dehydrogenase (GAPDH) in kidney homogenates of OXYS rats. Despite their role as housekeeping proteins, the COX IV levels may represent the work of the respiratory electron transport chain and indirectly indicate the mitochondrial content, as well as GAPDH may reflect the glycolytic activity.

**Figure 4:**
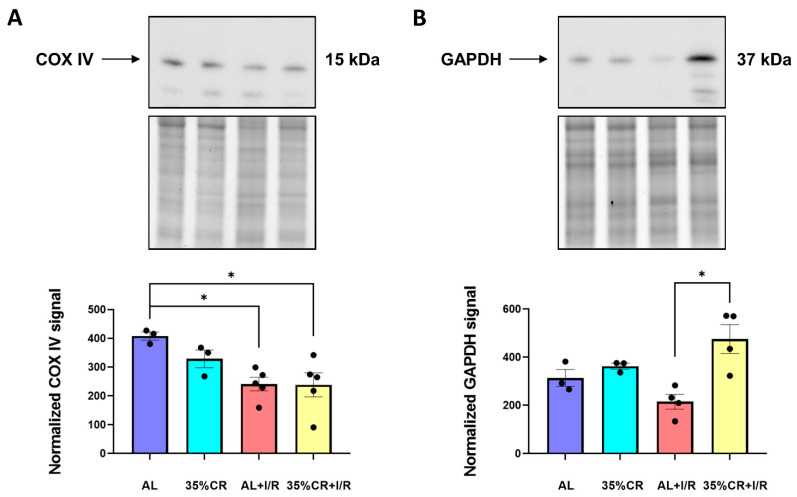
The levels of proteins of respiratory electron transport chain and glycolysis. (**A**) The levels of COX IV in kidney mitochondria of OXYS rats on 35% CR or AL diet, before and 48 h after renal I/R (*n* = 3 for AL group, *n* = 3 for 35% CR group, *n* = 5 for AL+I/R group, *n* = 5 for 35% CR+I/R group); (**B**) The level of GAPDH in kidney homogenates of OXYS rats on 35% CR or AL diet, before and 48 h after renal I/R (*n* = 3 for AL group, *n* = 3 for 35% CR group, *n* = 4 for AL+I/R group, *n* = 4 for 35% CR+I/R group). Below the blot image of the target protein, the corresponding total protein loading estimated by Stain-Free imaging technique is presented. * *p* < 0.05 (one-way ANOVA).

We observed that under I/R conditions the content of COX IV decreased significantly in OXYS rats kept both on the AL and CR diets (Figure 4A). Such a decrease in the COX IV levels may indicate the response of cells to the lack of oxygen and nutrient deficiency that occurred 48 h earlier. The GAPDH level also changed in the kidneys of OXYS rats subjected to CR, especially 48 h after I/R (Figure 4B). Of note, the level of another housekeeping protein, β-actin was also significantly reduced after I/R, in OXYS rats kept either on CR or AL diet (Supplementary Figure S3). Total protein visualization performed by the Stain-Free imaging technique or Coomassie Blue staining showed equal protein loading in all samples (Supplementary Figure S4).

### 2.5. CR Does Not Activate Autophagy in OXYS Rats but Causes Signs of Mitochondria Renewal

During ischemic injury or fasting, autophagy is one of the mechanisms regulating the balance of the intracellular protein composition, as well as the maintenance of cellular organelles functioning [44]. Autophagy activation counteracts the age-related accumulation of damaged cellular components and increases the metabolic efficiency of cells [45]. To elucidate a possible change in the level of autophagy in response to CR in OXYS rats, we measured the levels of LC3 protein. Activation of autophagy causes post-translational modification of this protein. Ultimately, LC3 loses its C-terminal fragment and conjugates to phosphatidylethanolamine, transforming first into LC3 I and then into LC3 II. The LC3 II/I ratio directly correlates with the autophagic flux in the cell [46].

**Figure 5:**
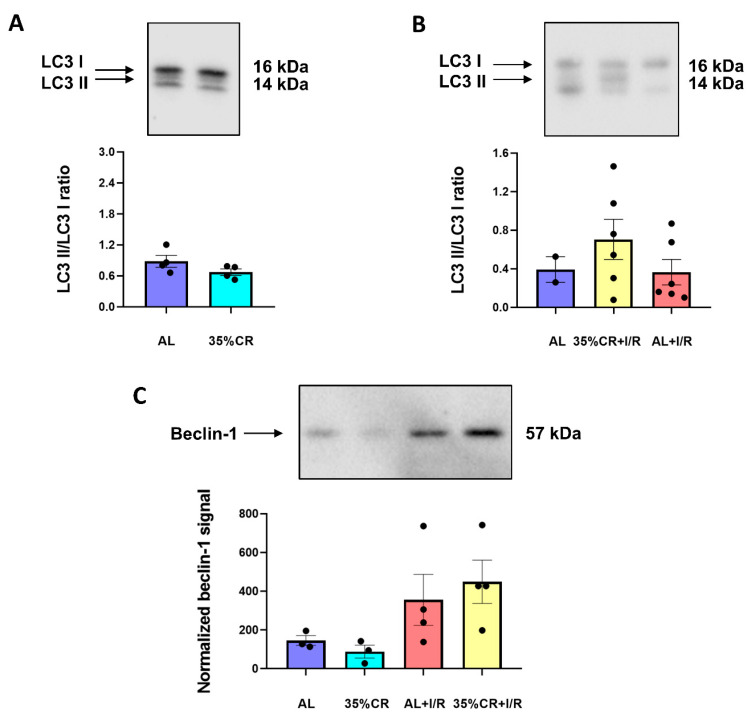
The absence of autophagy activation in kidney under different experimental protocols. (**A**) LC3 II/I ratio in kidney homogenates of OXYS rats on 35% CR or AL diet (*n* = 4 for AL group, *n* = 4 for 35% CR group); (**B**) LC3 II/I ratio in kidney homogenates of intact rats and 48 h after renal I/R, in OXYS rats kept on AL or 35% CR diet (*n* = 2 for AL group, *n* = 6 for 35% CR+I/R group, *n* = 6 for AL+I/R group)**;** (**C**) Beclin-1 in kidney homogenates of OXYS rats on 35% CR or AL diet, before and 48 h after renal I/R (*n* = 3 for AL group, *n* = 3 for 35% CR group, *n* = 4 for AL+I/R group, *n* = 4 for 35% CR+I/R group).

In kidney homogenates of OXYS rats, 35% CR for 4 weeks did not cause the increase in LC3 II/I ratio indicating the absence of autophagy activation (Figure 5A). We also evaluated LC3 II/I ratio in kidneys of OXYS rats 48 h after I/R and observed the lack of autophagy activation (Figure 5B). As an additional marker of autophagy activation, we analyzed the levels of beclin-1, which is a core protein of autophagy machinery upregulating in different pathologies, including renal I/R [47]. We observed that beclin-1 tended to increase 48 h after I/R in kidneys of OXYS rats (Figure 5C). However, CR did not change the levels of this autophagic protein in OXYS rats, corroborating the data on LC3 II/I ratio and the lack of autophagy activation.

**Figure 6:**
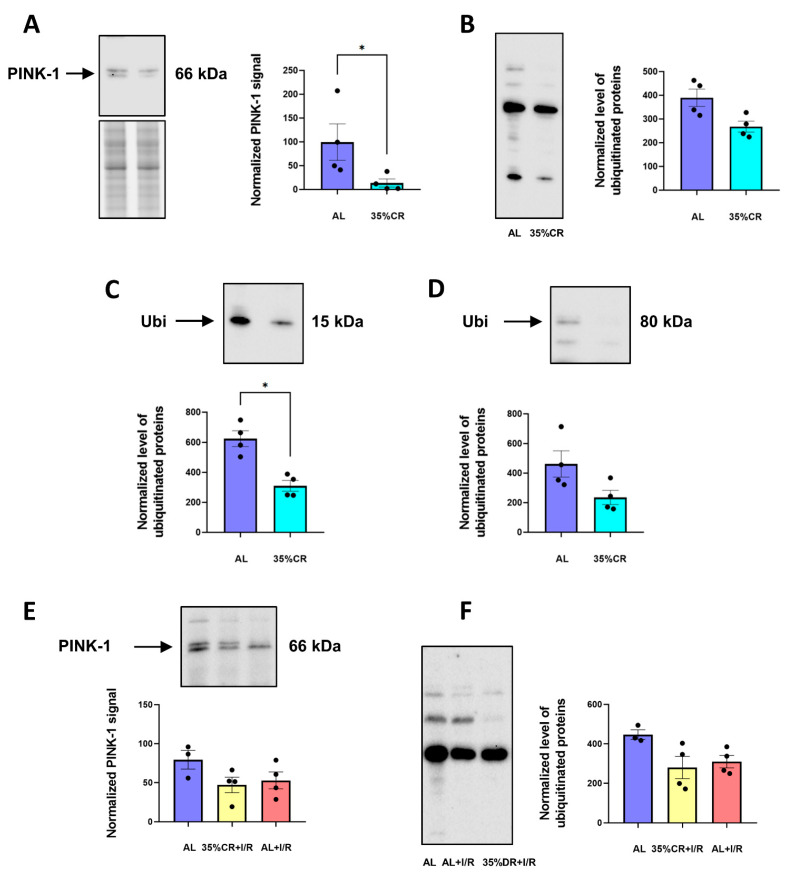
Evaluation of mitophagy activation in the kidneys. (**A**) PINK-1 level in isolated kidney mitochondria of OXYS rats on 35% CR or AL diet (*n* = 4 for AL group, *n* = 4 for 35% CR group); (**B**) Total ubiquitination in isolated kidney mitochondria of OXYS rats kept either on 35% CR or AL diet (*n* = 4 for AL group, *n* = 4 for 35% CR group); (**C**,**D**) Ubiquitination level in proteins with approximate molecular masses of 15 kDa and 80 kDa in isolated kidney mitochondria of OXYS rats on 35% CR or AL diet (*n* = 4 for AL group, *n* = 4 for 35% CR group); (**E**) PINK-1 level in isolated kidney mitochondria from intact OXYS rats and from those 48 h after renal exposure to I/R, kept either on AL or CR diet (*n* = 3 for AL group, *n* = 4 for AL+I/R group, *n* = 4 for 35% CR+I/R group); (**F**) Total ubiquitination in isolated kidney mitochondria of intact OXYS rats and 48 h after renal I/R, in OXYS rats kept on AL or CR diet (*n* = 3 for AL group, *n* = 4 for AL+I/R group, *n* = 4 for 35% CR+I/R group). Below the blot image of the target protein, the corresponding total protein loading estimated by Stain-Free imaging technique is presented (**A**). * *p* < 0.05 (Mann–Whitney U-test).

Normally, autophagosomal removal of mitochondria, which is called mitophagy, occurs in cells at a constant low rate and first of all utilizes poorly functional mitochondria [48]. This rate can be increased, for instance, during CR [49]. Mitophagy is a complex multicomponent process, the key role in which is played by PTEN-induced putative kinase 1 (PINK-1). Interaction between PINK-1 and PARKIN causes polyubiquitination of the mitochondrial proteins, recruitment of autophagic receptors, and further autophagosomal mitochondrial degradation. In isolated mitochondria of OXYS rats, we measured the PINK-1 level and the content of ubiquitinated proteins using western blotting (Figure 6). CR decreased PINK-1 content on the mitochondria indicating less proportion of mitochondria with low membrane potential. After I/R, the level of PINK-1 did not differ in rats on AL and CR diets (Figure 6E). 

The changes in PINK-1 levels correlated with a tendency towards a decrease in the total ubiquitination level of all detectable proteins in mitochondria from OXYS rats in the CR group (Figure 6B). The most significant drop of ubiquitination was detected for proteins with approximate molecular mass in 15 kDa and 80 kDa (Figure 6C,D). Similar to PINK-1 levels, total mitochondrial protein ubiquitination after I/R was the same in rats on AL or CR diet (Figure 6F).

We also examined the mitochondrial dynamics pathways in terms of the levels of the proteins participating in mitochondrial fusion and fission. Mitochondrial dynamics regulates the mitochondrial network and contributes to the mitochondrial function and quality control [50]. We evaluated the levels of long and short (cleaved) forms of mitochondrial fusion protein OPA1, which tended to increase after I/R (Supplementary Figure S5A,B). We also analyzed the levels of mitochondrial fission proteins Fis1 (Supplementary Figure S5C) and Drp1 (Supplementary Figure S5D). Drp1 demonstrated a significant increase 48 h after I/R, proving the prevalence of mitochondrial fission during I/R, with CR normalizing this process in kidneys of OXYS rats (Supplementary Figure S5D). 

### 2.6. CR Effects on Protein Acetylation and Cellular Senescence in OXYS Rats

**Figure 7:**
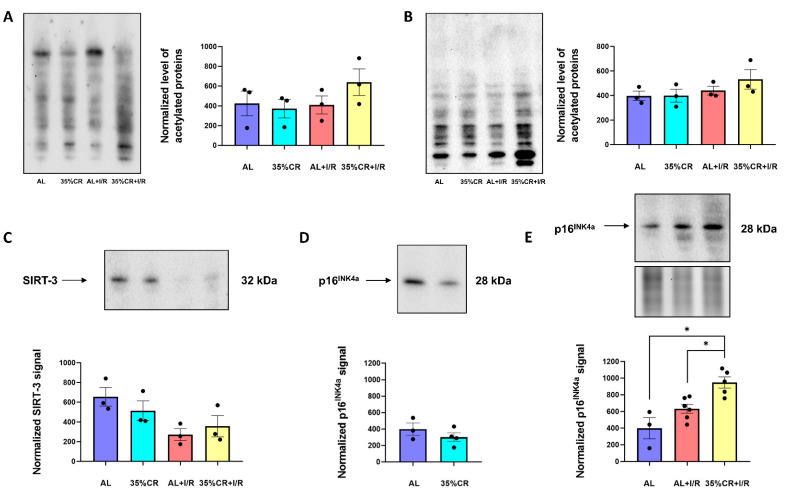
Acetylation profile and the levels of senescence markers. (**A**) Total acetylation in kidney homogenates of OXYS rats kept on 35% CR or AL diet, before and 48 h after renal I/R (*n* = 3 for AL group, *n* = 3 for 35% CR group, *n* = 3 for AL+I/R group, *n* = 3 for 35% CR+I/R group); (**B**) Total acetylation in isolated kidney mitochondria of OXYS rats on 35% CR or AL diet, before and 48 h after renal I/R (*n* = 3 for AL group, *n* = 3 for 35% CR group, *n* = 3 for AL+I/R group, *n* = 3 for 35% CR+I/R group); (**C**) The level of SIRT-3 in isolated kidney mitochondria of OXYS rats on 35% CR or AL diet (*n* = 3 for AL group, *n* = 3 for 35% CR group, *n* = 3 for AL+I/R group, *n* = 3 for 35% CR+I/R group); (**D**) p16^INK4a^ level in isolated kidney mitochondria of OXYS rats on 35% CR or AL diet (*n* = 3 for AL group, *n* = 4 for 35% CR group); (**E**) p16^INK4a^ level in isolated kidney mitochondria of intact OXYS rats and 48 h after renal I/R, in OXYS rats kept on AL or CR diet (*n* = 3 for AL group, *n* = 6 for AL+I/R group, *n* = 5 for 35% CR+I/R group). Below the blot image of the target protein, the corresponding total protein loading estimated by Stain-Free imaging technique is presented (**E**). * *p* < 0.05 (one-way ANOVA).

Reduced calorie intake leads to a decreased acetyl-CoA concentration, ATP production, and ratio of redox NADH/NAD^+^ couple [51]. As a result, many proteins may undergo the change in their post-translational modifications, particularly in their acetylation profile. Acetylation is a type of post-translational protein modification and it belongs to robust intracellular regulatory factors. For instance, the lack of acetyl-CoA and further deacetylation of various proteins, including proteins of the autophagic system, promotes autophagy [52]. In kidney homogenates of OXYS rats, the effects of CR and I/R on total protein acetylation in the molecular mass range from 9 to 200 kDa were small and highly variable; the averaged data for all groups showed no statistical difference (Figure 7A). In isolated kidney mitochondria, there was also no significant difference in total acetylation levels (Figure 7B). 

The level of protein acetylation is also strongly related to the activity of acetyltransferases and deacetylases. Among the families of these enzymes, NAD+-dependent deacetylases (sirtuins) play a unique role [53]. Sirtuins require NAD+ as a cofactor so they act as sensors of the amount of nutrients in cells. SIRT-3 is the most abundant sirtuin in mitochondria and it regulates the activity of mitochondrial proteins involved in metabolism and oxidative stress-related signaling pathways [54]. In our study, we evaluated the level of SIRT-3 in isolated kidney mitochondria of OXYS rats. Surprisingly, SIRT-3 did not increase in OXYS rats in response to CR. Moreover, there was some tendency towards a decrease of this deacetylase content (Figure 7C).

We also evaluated the effects of CR on cellular senescence p16^INK4a^, a principal mediator of cellular senescence, whose expression markedly increases in almost all rodent tissues during aging [55]. We did not observe any decrease in p16^INK4a^ level in kidney tissue of OXYS rats after CR (Figure 7D). However, we showed a significant increase in p16^INK4a^ levels 48 h after I/R in 35%CR+I/R group (Figure 7E), possibly indicating more pronounced activation of quality control system and cell proliferation.

## 3. Discussion

In this study, we analyzed the protective properties of CR against ischemic AKI in senescence-accelerated OXYS rats. Our main finding was that at the physiological level, 35% CR for 4 weeks prior to renal I/R caused significant nephroprotective effects in senescence-accelerated OXYS rats (Figure 2). We also explored some signaling pathways that are normally associated with a positive effect of CR in other strains of rats. Such analysis enabled us to evaluate the possible mechanisms of nephroprotection provided by CR in OXYS rats and to compare the physiological responses of OXYS rats and Wistar rats of different ages.

In general, the protective properties of CR have been shown for a long time among a vast variety of animal species [56]. CR is one of the few approaches that significantly increase the life expectancy of various animals from yeast to apes [57]. It has been shown that its numerous effects are mainly expressed in slowing down age-related diseases, including myocardial infarction [58], stroke [59], hypertension [60], neurodegenerative diseases such as Alzheimer’s disease [61], metabolic syndrome and diabetes mellitus [62,63], and cancer [64]. It is believed that such a reduction in food consumption by 30–40% does not lead to the crossing of the minimum mandatory consumption of essential nutrients [11].

AKI also refers to age-associated diseases since the majority of patients with AKI are elderly people [65]. Numerous studies have already demonstrated significant nephroprotective effects of CR in various kidney diseases. In young animals, the severity of the renal injury was reduced even after relatively short-term CR [66,67]. In our previous studies, we have also revealed that in young rats, 35% CR for 4 weeks lowered the severity of AKI, reducing the concentrations of SCr and BUN more than 3-fold [68,69].

In old animals, 35% CR for 4 or 8 weeks was less effective in kidney tissue and caused positive changes only at the molecular, but not physiological level [68,69]. In old animals, CR efficacy may be reduced and longer CR periods are potentially required. The decrease in CR efficiency during aging can be explained by morphological and cellular effects on the kidney tissue [70]. At the molecular level, even relatively short periods of CR still lead to improved kidney function in old animals [71,72]. Moreover, life-long CR can even slow down the development of age-associated renal histological abnormalities and age-related chronic nephropathy [73,74].

Most of the CR effects are accomplished through several metabolic and stress-resistance pathways, affecting primarily the metabolism of nutrients. The main signaling pathways that are activated during CR are the decreased response from insulin-like receptors, activation of AMPK, and NAD+-dependent deacetylases sirtuins [75]. Acting through mTOR complexes, FoxO and CREB transcription factors, a decrease in nutrient intake and the activation of the above-mentioned pathways inevitably affect the functioning of mitochondria [76]. CR leads to an improvement in mitochondrial structure and functions in many organs [77], in particular, by promoting their biogenesis, as well as maintaining mitochondrial network homeostasis and functional coordination with peroxisomes to increase fatty acid oxidation [78]. Thus, it is accepted at least in part that the longevity-promoting effects of CR can be modulated by mitochondrial and antioxidant activities [13].

It is essential to maintain a population of properly functioning mitochondria, especially prior to ischemic injury since mitochondria play a crucial role in this type of injury [38]. Damaged mitochondria are incapable of providing efficient energy synthesis, fatty acid oxidation, and biosynthesis of amino acids and maintaining adequate intracellular calcium and iron metabolism [79]. Decreased ATP production, excessive ROS generation, and a violation of calcium exchange between mitochondria and the cytosol, all induce the opening of mitochondrial permeability transition pore (mPTP), which leads to depolarization of the inner mitochondrial membrane [80]. All these processes inevitably lead to damage to the mitochondria themselves and other organelles and macromolecules or cause cell death.

With age, mitochondria functioning deteriorates. Thereby, altered mitochondrial metabolism is a well-known hallmark of aging [81]. Aging is associated with a loss of mitochondrial network homeostasis, genomic instability, impairment of mitochondrial communication with other organelles, and excessive ROS production. This occurs gradually by a slow accumulation of damaged molecules in mitochondria due to the influence of external and intrinsic stress factors [82]. Mitochondrial dysfunction can be a major cause of a wide range of age-related diseases, including AKI [83].

In this study, we demonstrate that the improvement of mitochondrial functioning may be responsible for the main effects of CR in senescence-accelerated OXYS rats among all other considered mechanisms. In OXYS rats, we observed a significant decrease in COX IV level, which may be associated with disrupted work of the respiratory electron transport chain and reduced number of functional mitochondria 48 h after I/R (Figure 4A). A similar decrease in COX IV level was noticed as a result of stroke when the activities of all complexes of the respiratory electron transport chain were inhibited [84,85]. Remarkably, CR prevented the decrease in the COX IV level during I/R and returned the level of this protein to the values of intact animals (Figure 4A). In OXYS rats, we also detected the CR-associated changes in anti-apoptotic Bcl-X_L_ protein and pro-apoptotic Bcl-X_S_ form, especially after I/R (Figure 3 and Figure S2). Such changes may increase the tolerance of kidney tissue to injury and the resistance of cells to apoptosis.

We also analyzed the content of GAPDH, which functions are not limited by its housekeeping role. Initially, GAPDH was identified as a protein involved in glycolysis [86]. Recently, it has become clear that the role of this protein in the cell is much more complex and may vary in a much higher range than was previously thought. In addition to its role in glycolysis, GAPDH is also involved in the repair process and control of protein expression [87]. An increase in GAPDH level after I/R in OXYS rats kept on CR diet (Figure 4B) may undermine both increased glycolytic flux as a compensatory cellular response to ischemia and higher participation of GAPDH in DNA repair or other effects on protein expression after injury [88].

Mitochondrial dynamics maintains the mitochondrial network, contributes to the mitochondrial function and quality control, and regulates mitochondrial morphology, number, and distribution within the cell [89]. The balance between mitochondrial fusion and fission processes is important for mitochondrial bioenergetics [90]. To date, there is very limited data about the patterns of mitochondrial dynamics in kidneys during CR. In general, it is supposed that CR leads preferentially to mitochondrial fusion, which has a protective effect on mitochondrial DNA recovery and delaying apoptosis [91] while inhibition of the mitochondrial fusion/fission process specifically blocked CR-mediated longevity [92]. In this regard, in our study we analyzed the levels of the main proteins participating in mitochondrial dynamics. We showed that the processes of mitochondrial fission rather than fusion dominated in kidneys of OXYS rats after renal I/R with CR partially normalizing this proportion (Supplementary Figure S5).

The most important function of mitochondria is to maintain an energy level in the cell. Acetyl-CoA, which is formed mainly during the metabolism of sugars and β-oxidation of fatty acids, is metabolized in the tricarboxylic acid cycle and provides the cell with energy and reduced forms of coenzymes NADH and NADPH. The amount of acetyl-CoA in the cell directly affects the protein acetylation profile [93]. In OXYS rats, CR did not significantly influence the level of total protein acetylation, whereas some proteins tended to change their acetylation (Figure 7A,B).

The maintenance of the acetylation profile in cells is partially achieved through the work of NAD+-dependent deacetylases sirtuins [53]. The levels of almost all sirtuins increase during CR, which may indicate the important role of these proteins in mediating the beneficial effects of CR and promoting longevity [13]. In our previous study, we showed that in the kidneys of young Wistar rats, 35% CR for 4 weeks led to a significant increase in the level of SIRT-3 in comparison with young Wistar rats kept on AL diet [69]. In contrast to young rats, in old animals even more prolonged CR did not lead to significant differences in the SIRT-3 level. Thus, OXYS rats by this parameter were more similar to old animals, since their SIRT-3 level did not increase (Figure 7C).

The improvement in mitochondrial function during CR is inextricably linked to the mitophagy process. Poorly functioning mitochondria having reduced efficiency of energy production, and simultaneously being a source of increased ROS become a subject for utilization by mitophagy [94]. However, with age, the precise quality control mechanism of mitochondria deteriorates [95]. Among the various stimuli for the onset of mitophagy, CR is one of the strongest non-genetic triggers for initiating the mitophagy process and improvement of mitochondrial quality control [49]. Mitochondrial quality control attenuates age-related declines in mitochondrial function and causes renewal of the mitochondrial network thereby slowing down aging and age-associated diseases [96], thus protecting the kidneys of old animals [97]. In this study, a trend towards the activation of mitophagy was noticed in senescence-accelerated OXYS rats subjected to CR.

In general, the activation of mitophagy is a particular manifestation of the entire autophagy process. The lack of nutrients strongly stimulates autophagy [49], utilizing first of all damaged macromolecules and organelles. We have previously shown that in young rats, 35% CR for 4 weeks strongly enhances autophagy, whereas in old animals the expected activation of autophagy was not found even after a more prolonged (8 weeks) CR [69]. In the current study, 35% CR for 4 weeks in senescence-accelerated OXYS rats did not lead to a significant increase in the LC3 II/LC3 I ratio or beclin-1 levels (Figure 5). Thus, in OXYS rats, CR did not cause activation of autophagy, which is a specific trait of old animals.

The mitochondrial function may also strongly regulate the level of oxidative stress. The accumulation of poorly functioning mitochondria is dangerous for cells as it can cause increased oxidative stress and disrupt the work of other organelles and macromolecules [98]. Since mitophagy is responsible for the utilization of such mitochondria, the deterioration of mitophagy with age is directly related to an increase in the severity of oxidative stress [99]. In our study, we showed that CR did not cause any reduction in the oxidative stress level in senescence-accelerated OXYS rats (Figure 3D,E), proving their senescent phenotype.

## 4. Materials and Methods

### 4.1. OXYS Rats

In this study, male 6-month-old OXYS rats (300–350 g) were used. The OXYS rat strain originating from Wistar outbred rats through selection for susceptibility to the cataractogenic effect of a galactose-rich diet and inbreeding was obtained from the Institute of Cytology and Genetics (Novosibirsk, Russia) [17]. Rats were used according to animal protocols evaluated and approved by the animal ethics committee of A.N. Belozersky Institute of Physico-Chemical Biology Lomonosov Moscow State University: Protocol 3/19 from 18 March 2019. All procedures were in accordance with the «Animal Research: Reporting of In Vivo Experiments» (ARRIVE) guidelines. Rats were randomly divided into the following experimental groups: AL (*n* = 4), 35% CR (*n* = 4), AL+I/R (*n* = 7), 35% CR+I/R (*n* = 6). Rats from AL and 35% CR groups were used as controls and were subjected only to the corresponding diet.

### 4.2. Dietary Protocol

The diet was standard for laboratory rodents and contained 19% proteins and 5% fat with calorie content 300 kcal/100 g of chow. The amount of food consumed by AL was approximately 22 g/rat per day (6.4 g/100 g of body weight per day), as measured by weighing the remaining food for two weeks. CR was performed for 4 weeks by limiting the amount of food by 35% of the normal daily intake. Food was administered daily once at 1:00 p.m. For all groups, free access to water was implemented. Weekly, each rat was weighed for monitoring changes in body mass.

### 4.3. Renal I/R Protocol

For modeling AKI, rats were subjected to 40-min warm ischemia of the left kidney. During the surgery procedure, the renal vascular bundle was occluded for 40 min with a microvascular clip for ischemia induction. During ischemia, the body temperature of the OXYS rats was maintained at 37 ± 0.5 °C with an automatic system equipped with infrared lamps. At the same time, microsurgical manipulations were performed on a thermostatically controlled heating mat. The control of body temperature was conducted to reduce the effect on AKI severity of lowering body temperature during general anesthesia. Simultaneously, with ischemia of the left kidney nephrectomy of the right one was performed for more pronounced renal function deterioration. After 40 min of ischemia, circulation of blood in the left kidney was restored by removing the microvascular clip. The lack of blood flow during ischemia and its restoration during reperfusion were assessed visually by the change of the kidney color. Further manipulations, including blood, kidney homogenates, and mitochondria samples preparation were performed 48 h after kidney I/R.

### 4.4. Western Blotting of Kidney Homogenates

Rats were sacrificed and kidneys were taken from control rats or 48 h after I/R, subjected to AL or CR diet. Samples of kidney tissue were homogenized with a glass-Teflon homogenizer in a PBS buffer containing 10 mM phenylmethylsulfonyl fluoride at 4 °C. The homogenate was centrifuged at 3000 rpm for 3 min, the supernatant was mixed with 4x sample buffer containing 10% 2-mercaptoethanol, and boiled for 5 min. Protein concentration was measured by bicinchoninic acid assay (Sigma, Burlington, MA, USA). Total protein loading was also controlled by the Stain-Free imaging technique according to the manufacturer’s instructions (#1610185, BioRad, Hercules, CA, USA). All western blot bands were normalized to the intensities of the same samples on Stain-Free images. As an additional approach for assessing the total protein loading, we also performed gel staining with Coomassie Blue (Helicon, Moscow, Russia).

Kidney samples were loaded onto 15% Tris-glycine polyacrylamide gels (10 μg protein/lane). After electrophoresis, gels were transferred onto PVDF membranes (Amersham Pharmacia Biotech, Buckinghamshire, UK). Membranes were blocked with 5% non-fat milk in PBS with 0.05% Tween-20 and subsequently incubated with primary antibodies: anti-KIM-1 1:1000 mouse (MAA785Ra21, Cloud Clone Inc., Wuhan, Hubei, PRC), anti-PCNA 1:1000 rabbit (#13110, Cell Signaling, Danvers, MA, USA), anti-Bcl-X 1:1000 rabbit (#2764, Cell Signaling, USA), anti-OPA1 1:1000 rabbit (ab42364, Abcam, Cambridge, UK), anti-COX IV 1:1000 mouse (#A21348, Invitrogen, Waltham, MA, USA), anti-GAPDH 1:1000 mouse (#5G4cc, Hytest, Turku, Finland), anti-b-actin 1:2000 mouse (#A2228, Sigma-Aldrich, USA), anti-LC3A/B 1:1000 rabbit (#12471, Cell Signaling, USA), anti-beclin-1 1:1000 rabbit (#3495, Cell Signaling, USA), anti-OPA1 1:1000 rabbit (ab42364, Abcam, UK), anti-acetylated-Lysine 1:1000 rabbit (#9441, Cell Signaling, USA), anti-p16INK4a 1:1000 rabbit (ab108349, Abcam, UK). Membranes were then incubated with secondary antibodies: anti-rabbit IgG or anti-mouse IgG conjugated with horseradish peroxidase 1:7500 (Jackson ImmunoResearch, West Grove, PA, USA) and probed with Advansta Western Bright ECL kit (Advansta, San Jose, CA, USA). Detection was performed by V3 Western Blot Imager (BioRad, USA). Carbonylated proteins were measured using the OxyBlot kit according to the manufacturer’s instructions (S7150, OxyBlot Protein Oxidation Detection Kit, Millipore, Danvers, MA, USA).

### 4.5. Mitochondria Isolation and Western Blotting

Mitochondria were isolated from kidneys of OXYS rats kept on the AL or CR diet. The rat kidney mitochondria for western blotting were isolated by differential centrifugation in the medium containing 0.25 M sucrose, 10 mM HEPES, 1 mM EDTA, 0.1% BSA, pH 7.4 [100]. All further steps correspond to those for western blotting of kidney homogenates. Total protein loading was estimated by Stain-Free imaging technique according to the manufacturer’s instructions (#1610185, BioRad, USA). Membranes were incubated with primary antibodies: anti-Bcl-X 1:1000 rabbit (#2764, Cell Signaling, USA), anti-COX IV 1:1000 mouse (#A21348, Invitrogen, USA), anti-PINK-1 1:1000 rabbit (ab23707, Abcam, UK), anti-ubiquitin 1:1000 rabbit (ab7780, Abcam, UK), anti-Fis1 1:1000 rabbit (ab71498, Abcam, UK), anti-Drp1 1:1000 mouse (611739, BD Biosciences, Franklin Lakes, NJ, USA), anti-acetylated-Lysine 1:1000 rabbit (#9441, Cell Signaling, USA), anti-SIRT3 1:1000 rabbit (#2627, Cell Signaling, USA). Similarly, as for kidney homogenates, membranes were then incubated with secondary antibodies: anti-rabbit IgG or anti-mouse IgG conjugated with horseradish peroxidase 1:7500 (Jackson ImmunoResearch, USA) and probed with Advansta Western Bright ECL kit (Advansta, USA). The concentration of total mitochondrial protein was measured with bicinchoninic acid assay (Sigma, USA). Carbonylated proteins were detected using the OxyBlot kit according to the manufacturer’s instructions (S7150 OxyBlot Protein Oxidation Detection Kit, Millipore, USA).

### 4.6. Biochemical Analysis and Western Blotting of Serum

To confirm AKI, blood samples were taken 48 h after I/R from the carotid artery to determine SCr and BUN. After 15 min storage at room temperature, the clot was removed by centrifugation at 2000× *g* for 5 min. The resulting serum was frozen and later analyzed for SCr and BUN concentrations using the AU480 Chemistry System (Beckman Coulter, USA) according to the manufacturer’s instructions.

For western blotting, serum was diluted in the sample buffer, and the standard protocol of western blotting in polyacrylamide gel was performed. Membranes were incubated with primary anti-GDF11 1:1000 mouse (sc-81952, Santa Cruz, Dallas, TX, USA) antibodies. All further steps of western blotting of serum were similar to those for western blotting of kidney homogenates or isolated mitochondria.

### 4.7. Statistics

Values are presented as mean ± SEM. For experiments with two experimental groups, comparisons were made using non-parametric Mann–Whitney U-test. For experiments with three or four experimental groups, comparisons were made using one-way ANOVA. Data was analyzed using Microsoft Excel software (version KB4011684, WA, USA) and GraphPad Prism (version 8, GraphPad Software Inc., San Diego, CA, USA).

## 5. Conclusions

In this study, we evaluated the effects of CR on ischemic AKI in senescence-accelerated OXYS rats and demonstrated its pronounced protective properties. We demonstrated that CR affected metabolic pathways, improved mitochondria functions, and led to mitochondria renewal in the kidneys of OXYS rats. However, CR did not affect the proliferative response to injury, did not reduce oxidative stress, did not activate autophagy or mitochondrial dynamics, and did not change the protein acetylation profile or cellular senescence. Thus, we suggest that the nephroprotective effects of CR in OXYS rats are accomplished mostly through the improvement of mitochondria-associated pathways rather than other molecular targets.

## Data Availability

The data that support the findings of this study are available from the corresponding author upon reasonable request.

## Supplementary Materials

The following supporting information can be downloaded at https://www.mdpi.com/article/10.3390/ijms232315224/s1.
