# Supplementary material for: Calorie Restriction Provides Kidney Ischemic Tolerance in Senescence-Accelerated OXYS Rats
Source: Int J Mol Sci. 2022 Dec 3;23(23):15224. doi: 10.3390/ijms232315224 (PMC9735762; doi:10.3390/ijms232315224)
Supplement: Supplementary file 1 [file ijms-23-15224-s001.zip › ijms-1914876-supplementary.pdf]

Supplementary Figures

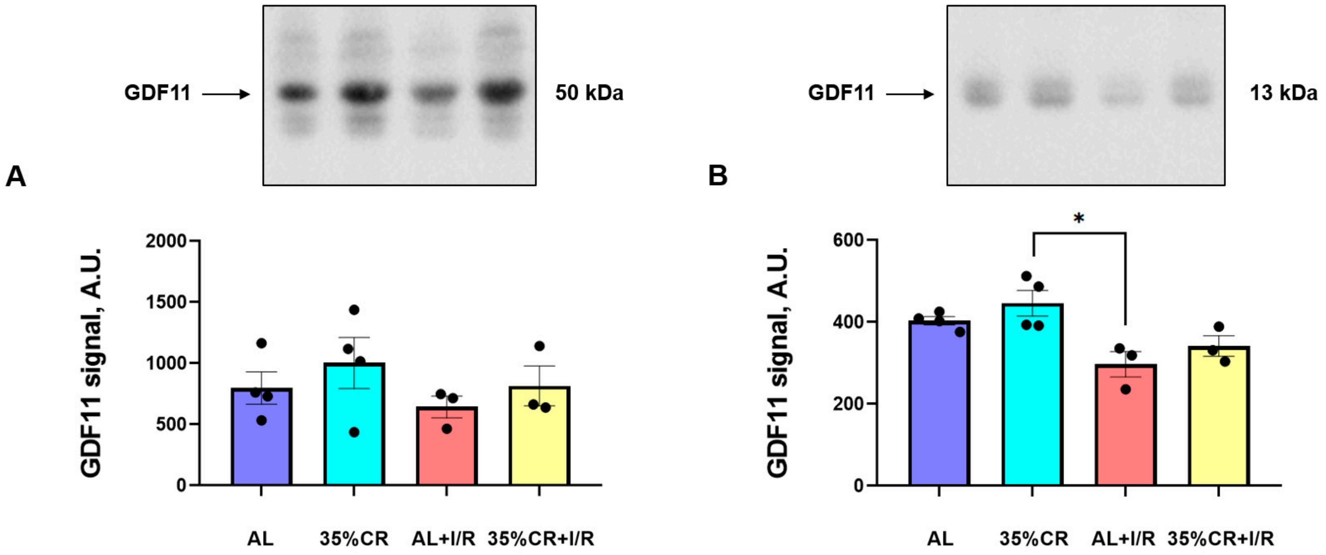

Supplementary Figure S1. The level of GDF11 in serum. **(A)** The level of precursor form of GDF11 in serum of OXYS rats on 35% CR or AL diet, before and 48 h after renal I/R (*n* = 4 for AL group, *n* = 4 for 35% CR group, *n* = 3 for AL+I/R group, *n* = 3 for 35% CR+I/R group). **(B)** The level of mature form of GDF11 in serum of OXYS rats on 35% CR or AL diet, before and 48 h after renal I/R (*n* = 4 for AL group, *n* = 4 for 35% CR group, *n* = 3 for AL+I/R group, *n* = 3 for 35% CR+I/R group). * $p < 0.05$ (one-way ANOVA).

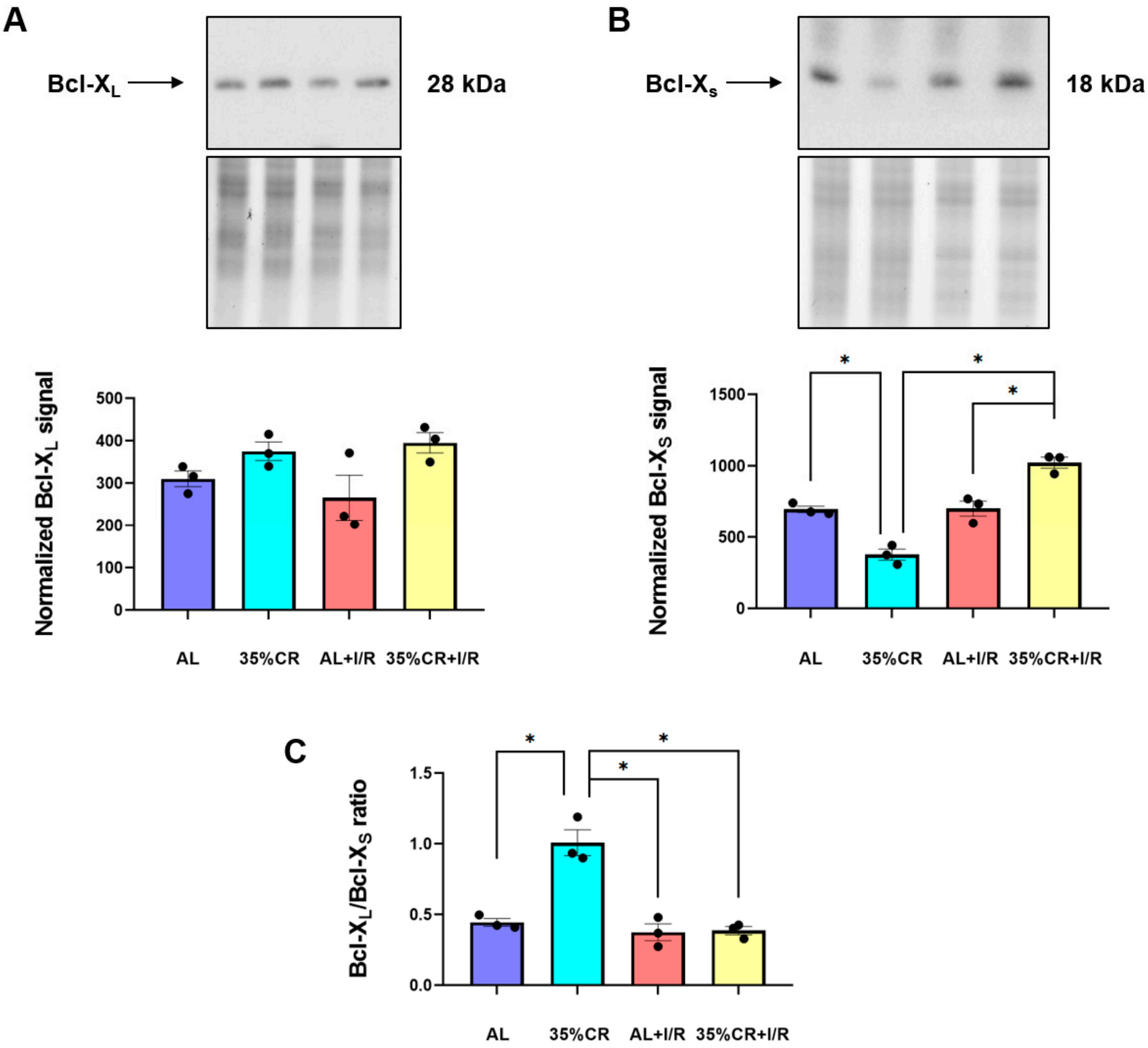

Supplementary Figure S2. The level of anti- and pro-apoptotic proteins in kidney homogenates. **(A)** Bcl-X_L levels in kidney homogenates of OXYS rats on 35% CR or AL diet, before and 48 h after renal I/R (n = 3 for AL group, n = 3 for 35% CR group, n = 3 for AL+I/R group, n = 3 for 35% CR+I/R group). **(B)** Bcl-X_S levels in kidney homogenates of OXYS rats on 35% CR or AL diet, before and 48 h after renal I/R (n = 3 for AL group, n = 3 for 35% CR group, n = 3 for AL+I/R group, n = 3 for 35% CR+I/R group). **(C)** Bcl-X_L/Bcl-X_S ratio in kidney homogenates of OXYS rats on 35% CR or AL diet, before and 48 h after renal I/R (n = 3 for AL group, n = 3 for 35% CR group, n = 3 for AL+I/R group, n = 3 for 35% CR+I/R group). Below the blot image of the target protein, the corresponding total protein loading estimated by Stain-free imaging technique is presented. * $p < 0.05$ (one-way ANOVA).

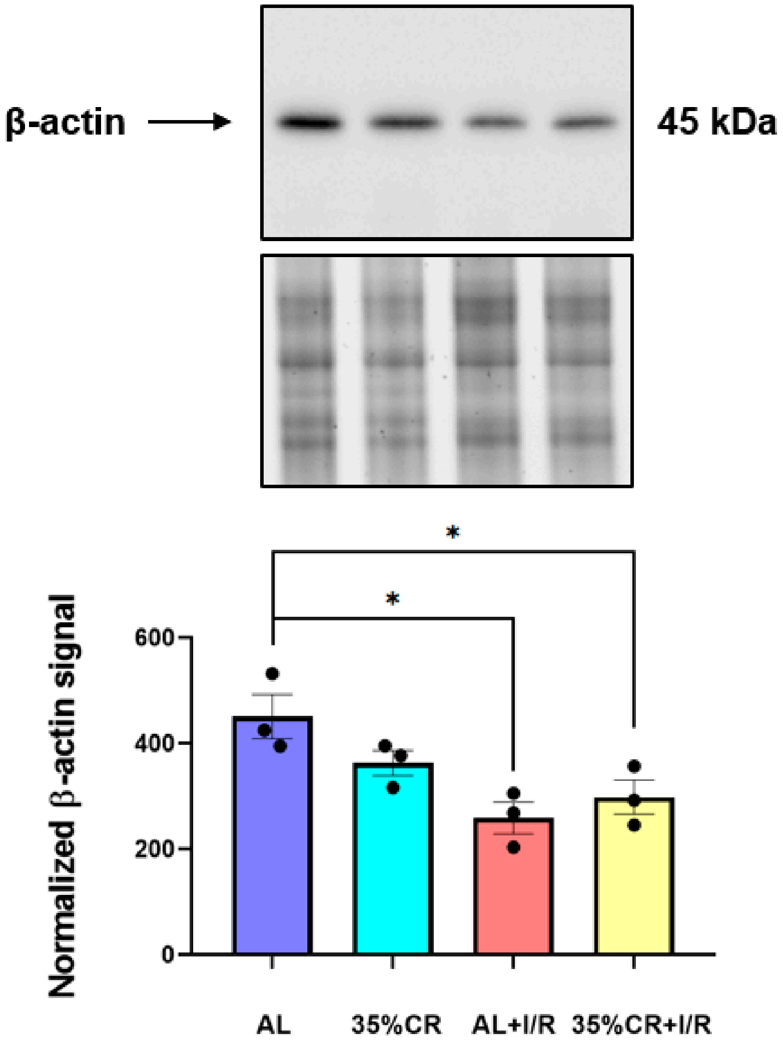

Supplementary Figure S3. The level of housekeeping protein β-actin in kidney homogenates of OXYS rats on 35% CR or AL diet, before and 48 h after renal I/R (n = 3 for AL group, n = 3 for 35% CR group, n = 3 for AL+I/R group, n = 3 for 35% CR+I/R group). Below the blot image of the target protein, the corresponding total protein loading estimated by Stain-free imaging technique is presented. * $p < 0.05$ (one-way ANOVA).

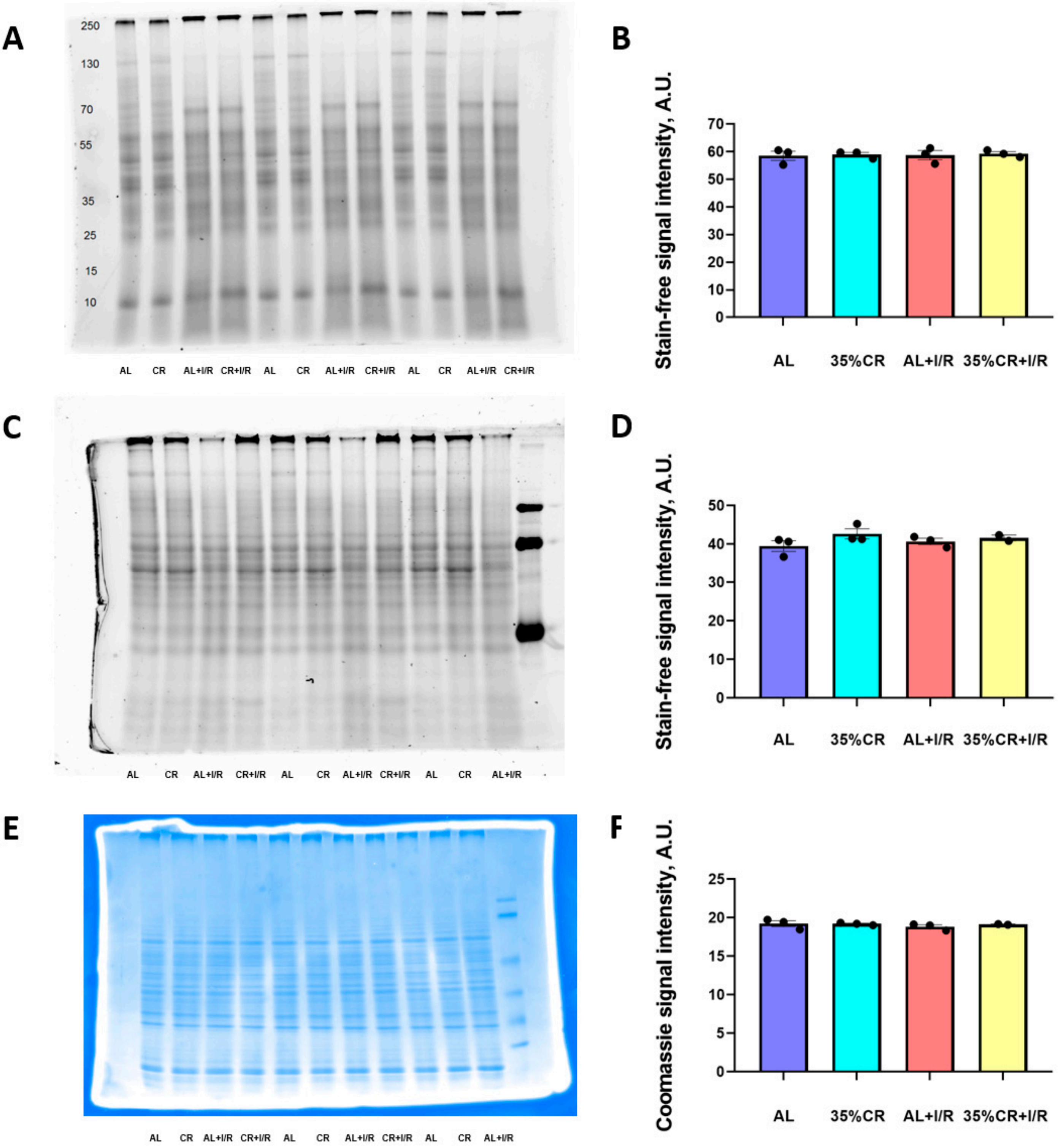

Supplementary Figure S4. Total protein normalization. **(A,B)** The example of Stain-free imaging of kidney homogenate samples (*n* = 3 for AL group, *n* = 3 for 35% CR group, *n* = 3 for AL+I/R group, *n* = 3 for 35% CR+I/R group). **(C,D)** The example of Stain-free imaging of isolated mitochondria samples (*n* = 3 for AL group, *n* = 3 for 35% CR group, *n* = 3 for AL+I/R group, *n* = 2 for 35% CR+I/R group). **(E,F)** Coomassie staining of kidney homogenate samples (*n* = 3 for AL group, *n* = 3 for 35% CR group, *n* = 3 for AL+I/R group, *n* = 2 for 35% CR+I/R group).

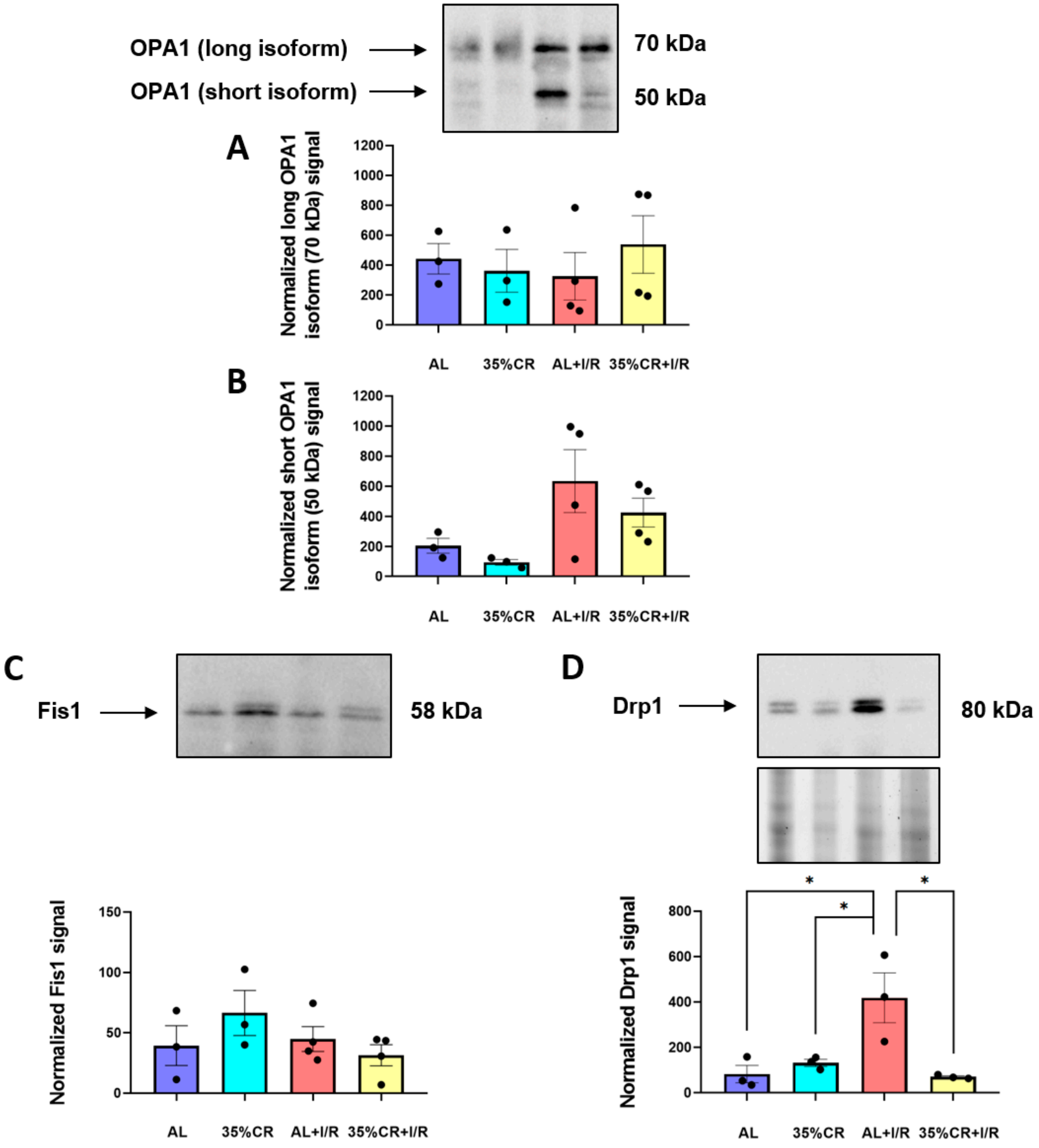

Supplementary Figure S5. The level of proteins participating in mitochondrial dynamics. **(A)** The level of long OPA1 isoform in kidney homogenates of intact OXYS rats and 48 h after renal I/R, in OXYS rats kept on AL or CR diet ($n = 3$ for AL group, $n = 3$ for 35% CR group, $n = 4$ for AL+I/R group, $n = 4$ for 35% CR+I/R group). **(B)** The level of short OPA1 isoform in kidney homogenates of intact OXYS rats and 48 h after renal I/R, in OXYS rats kept on AL or CR diet ($n = 3$ for AL group, $n = 3$ for 35% CR group, $n = 4$ for AL+I/R group, $n = 4$ for 35% CR+I/R group). **(C)** The level of Fis1 in isolated kidney mitochondria of intact OXYS rats and 48 h after renal I/R, in OXYS rats kept on AL or CR diet ($n = 3$ for AL group, $n = 3$ for 35% CR group, $n = 4$ for AL+I/R group, $n = 4$ for 35% CR+I/R group). **(D)** The level of Drp1 in isolated kidney mitochondria of intact OXYS rats and 48 h after renal I/R, in OXYS rats kept on AL or CR diet ($n = 3$ for AL group, $n = 3$ for 35% CR group, $n = 3$ for AL+I/R group, $n = 3$ for 35% CR+I/R group). * $p < 0.05$ (one-way ANOVA). Below the blot image of the target protein, the corresponding total protein loading estimated by Stain-free imaging technique is presented (D).
